# Is the Association between General Cognitive Ability and Violent Crime Caused by Family-Level Confounders?

**DOI:** 10.1371/journal.pone.0041783

**Published:** 2012-07-24

**Authors:** Thomas Frisell, Yudi Pawitan, Niklas Långström

**Affiliations:** 1 Department of Medical Epidemiology and Biostatistics, Karolinska Institutet, Stockholm, Sweden; 2 Centre for Violence Prevention, Karolinska Institutet, Stockholm, Sweden; George Mason University/Krasnow Institute for Advanced Study, United States of America

## Abstract

**Background:**

Research has consistently found lower cognitive ability to be related to increased risk for violent and other antisocial behaviour. Since this association has remained when adjusting for childhood socioeconomic position, ethnicity, and parental characteristics, it is often assumed to be causal, potentially mediated through school adjustment problems and conduct disorder. Socioeconomic differences are notoriously difficult to quantify, however, and it is possible that the association between intelligence and delinquency suffer substantial residual confounding.

**Methods:**

We linked longitudinal Swedish total population registers to study the association of general cognitive ability (intelligence) at age 18 (the Conscript Register, 1980–1993) with the incidence proportion of violent criminal convictions (the Crime Register, 1973–2009), among all men born in Sweden 1961–1975 (N = 700,514). Using probit regression, we controlled for measured childhood socioeconomic variables, and further employed sibling comparisons (family pedigree data from the Multi-Generation Register) to adjust for shared familial characteristics.

**Results:**

Cognitive ability in early adulthood was inversely associated to having been convicted of a violent crime (β = −0.19, 95% CI: −0.19; −0.18), the association remained when adjusting for childhood socioeconomic factors (β = −0.18, 95% CI: −0.18; −0.17). The association was somewhat lower within half-brothers raised apart (β = −0.16, 95% CI: −0.18; −0.14), within half-brothers raised together (β = −0.13, 95% CI: (−0.15; −0.11), and lower still in full-brother pairs (β = −0.10, 95% CI: −0.11; −0.09). The attenuation among half-brothers raised together and full brothers was too strong to be attributed solely to attenuation from measurement error.

**Discussion:**

Our results suggest that the association between general cognitive ability and violent criminality is confounded partly by factors shared by brothers. However, most of the association remains even after adjusting for such factors.

## Introduction

Although still somewhat controversial, it has long been recognized that there is an association between general cognitive ability (IQ) and criminal behaviour or delinquency [1]. The association has been found for both self-reported and officially recorded crime [2], [3], and appears stronger for repeat offending and violent or other severe types of crime [4]–[6]. Cognitive ability is often assumed to have a causal effect on criminal propensity, potentially mediated by school adjustment or performance [1], [7]–[9]. Others remain agnostic, arguing that the association may also be due to reverse causation, where low intelligence may be a consequence of conduct-disordered children’s truancy and lower education, or poorer motivation or attention during IQ-testing [10]. Arguments favouring the *low IQ causes crime* hypothesis focus on findings that low childhood intelligence is correlated to later adolescent delinquency and adult violence [3], [11]–[13], and some evidence that the IQ-delinquency association is attenuated by adjusting for school performance [7], [8], [12]. However, the latter argument should be interpreted cautiously, since conditioning on a mediating variable will introduce bias unless the association of mediator and outcome is completely causal [14], which is unlikely for the association of school performance and delinquency. Further, and contrary to the IQ → school factors → crime hypothesis, there is not only an association between pre-school IQ and adult criminality; there is also a simultaneous association of pre-school IQ and child conduct disorder [15], [16], a known precursor of adult antisocial behaviour.

The discussion of how cognitive ability influences criminal behaviour, or how criminal behaviour may influence cognitive ability, assumes that the association is not spurious; that is, confounded by some other factor(s). Initially, this claim was quite controversial, but after several studies reporting that the association remain when controlling for sex, race, and childhood socioeconomic position [17]–[20], and perhaps with growing acceptance of intelligence as a construct, the debate seems to have faded out. These early studies may be criticized, however, since they used limited measures of socio-economy, and made parametric assumptions that may not be correct (such as a linear effect of a 7-category measure of father’s occupation [17]). There is a substantial potential for residual confounding by socioeconomic factors, and other potential confounders such as parenting practices have not been controlled for explicitly.

An alternative to adjusting for measured confounding variables is the sibling comparison design. Siblings share many early socioeconomic variables, parental characteristics and rearing practices. Hence, any association between cognitive ability and criminal offending within families must be due to some other factor, or a sign of a causal association. We are only aware of two sibling comparisons addressing the association of cognitive ability and criminal offending. The first, published in 1936, compared 105 delinquents with their same-sex, nondelinquent siblings [21]. The second, based on 411 13-year-old twins recruited in London, is more recent but still quite small [22]. Although the authors did not seem to realize the implications, they analyzed the difference in twin-pair intelligence as a predictor for the difference in twin-pair behavioural deviance score, a design sometimes named the sibling difference design, and known to produce the same results as an ordinary between-within sibling comparison [23], [24]. Neither study found a statistically significant association of intelligence and offending within families. If this null finding were not due to poor statistical power, it would argue against a causal hypothesis.

In the present study, we elaborate on previous attempts at controlling for early childhood environmental differences. We used longitudinal Swedish total population registers to 1) estimate the association of cognitive ability and violent crime, 2) adjust the association for measured childhood socioeconomic factors, and 3) conduct sibling comparisons using a data set a thousand times larger than the two previous studies using sibling controls. The sample size enabled us to focus on convictions of violent crime, an outcome with even stronger public health importance than the more prevalent non-violent antisocial behaviour.

## Methods

To obtain information on relatedness, cognitive ability and criminal convictions for all men born in Sweden 1961–1975, we linked several nationwide Swedish registers. The Multi-Generation Register provided information on parents for all individuals born since 1932, and living in Sweden at any time after 1961. In the present cohort, register coverage was excellent; ca 1% had missing information on one or both parents. The Conscription Register contains information on psychological and physical characteristics of all men conscripted in Sweden. Conscription at age 18–20 was mandatory for all Swedish men until 2007; absence was a punishable offence. In the 1990s, less than 5% did not enlist, usually due to somatic illness or mental retardation [25]. The Crime Register covers all convictions in lower court from 1973 and onwards. Crimes are registered even when the sentence is non-custodial or involves forensic psychiatric treatment due to medico-legal insanity. Plea bargaining is not allowed in the Swedish judicial system. Finally, the Swedish age of criminal responsibility is 15 years; no crimes committed before this age are recorded in the Crime register.

The Cause of Death and Migration Registers provided information on deaths and emigration during follow-up, and information on socioeconomic characteristics was retrieved from the 1970 and 1975 national censuses. Each household had a unique identifier in the census, used here to ascertain if brothers were living together or apart. The household identifier in 1970 was used if both siblings were born before 1970 (i.e., 1961–1969), otherwise, data from 1975 were used.

### Ethics Statement

The study was approved by the Regional Ethics Committee in Stockholm, decision reference number 2009/939-31/5.

### Study Population

Of all men born in Sweden 1961–1975 (N = 874,388), we excluded individuals who had not undergone conscription at all (N = 98,641), those who had not undergone conscription 1980–1993 (N = 49,997), who lacked information on mother and/or father (N = 7690), general cognitive ability (N = 6584), or childhood residence (N = 10,962). This resulted in a study population of 700,514 men. A total of 10,813 (1.5%) individuals died and 42,599 (6.1%) emigrated before the end of follow-up; these were not excluded from the analyses. By focusing on men born in Sweden, we implicitly excluded Swedish residents born 1961–1975 in other countries (N = 270,575), since the immigrant group often lacked information on parents and, frequently, had not been conscripted.

Among these men, we identified 120,125 families with full-brothers (119,195 raised together [T]; 930 raised apart [A]), and 21,551 families with half-brothers (8474 maternal half-brothers T, 1875 maternal half-brothers A, 503 paternal half-brothers T, and 10,699 paternal half-brothers A). We then randomly selected one pair of brothers from each family; excluded full-brothers raised apart and grouped half-brothers by whether they were raised together. Thus, we analysed 238,390 full-brothers, 17,594 half-brothers T, and 25,148 half-brothers A.

### Outcome

The outcome was defined as having been convicted of one or more violent offences 1973–2009. We defined as “violent” any offence containing non-sexual interpersonal violence with the intention of physically or psychologically harming or coercing another individual. In line with previous studies [26], [27] we included homicide, assault, robbery, threats and violence against an officer, gross violation of a person’s/woman’s integrity, unlawful coercion, unlawful threat, kidnapping, illegal confinement, arson, and intimidation. We did not incorporate sexual offences (e.g. rape and child molestation), since they might differ etiologically from non-sexual violent offending [28]–[30]. Attempted and aggravated versions were included whenever applicable. For a description of the crimes above, see the online supplement of Frisell et al. (2011) [26].

### Main Exposure

General cognitive or intellectual capacity was measured by the Swedish Enlistment Battery used as part of the compulsory conscription 1980–1993 (SEB80). The SEB80 was developed to be psychometrically superior to the previous enlistment battery (SEB67; used 1967–1979), aimed at testing technical aptitude rather than general cognitive ability [25]. The SEB80 consisted of four subscales, originally aimed at capturing different aspects of cognitive ability (verbal, spatial, inductive, and technological). However, validation studies showed that, while the overall tests score was a good measure of general cognitive ability (*g*) or fluid intelligence (G*f*), the test could not reliably estimate lower order intelligence factors [31]. These studies, and the advent of personal computers, led to SEB80 being replaced in 1994, with a new test better suited at estimating also crystallized (G*c*) and general visualisation (G*v*) intelligence [32]. Intelligence was measured on a stanine scale (a normal distribution divided into nine categories, with a mean of 5 and standard deviation of 2). The scale was standardized each conscription year, so there was no change in the distribution over time.

We are not aware of any publically available reliability tests of the SEB80, but there are some figures for its predecessor SEB67. A subset of all men enlisting in 1965 were retested 1–4 years later, yielding high test-retest correlations (1 or 2 yrs = 0.89, 3 yrs = 0.80, 4 yrs = 0.84) [33]. Considering the psychometric improvement of the SEB80, we estimate the test-retest reliability of SEB80 to be at least similar.

### Potential Confounders

From the National Censuses of 1970 and 1975, we obtained socioeconomic characteristics of the households in which our study population lived at that time. Income was based on the taxed income of the “head of household” assigned in the census (in married couples, always the man). To overcome skewness and inflation, income was rank coded in deciles (1–10) in each census year. Living with a single mother was dichotomized and urbanicity was coded as a 10-category ordinal variable based on the population size of the urban area where the household was situated (<200; 200–499; 500–999; 1000–1999; 2000–4999; 5000–9999; 10,000–19,999; 20,000–49,999; 50,000–99,999; and ≥100,000 inhabitants).

### Statistical Analysis

The association of cognitive ability and violent criminal offending was analysed with probit regression using PROC GENMOD in SAS v9.2. Probit regression was selected since it provided significantly better fit to the data than the more commonly used logistic regression. The three types of brothers had slightly different birth year distributions (not shown), and birth year was directly associated with time-at-risk. To improve comparability across groups, all analyses adjusted for birth year as a categorical variable. We further adjusted for childhood socioeconomic factors: income, living with a single mother and urbanicity, all included as categorical variables. Missing rates where low for these covariates, only 8476 individuals in the full-brother sample, 677 in the half-brother T sample, and 783 individuals in the half-brother A sample missed information on one or more variables. These individuals were excluded from all regression models. Confidence intervals were based on robust standard errors, to account for the correlation of brothers in each pair. To adjust for unmeasured confounders shared by brothers, we performed a sibling comparison in a between-within model. This model is a simple extension of the regression described above, with the pair’s mean cognitive ability included as a covariate in the model. It has been shown that in the absence of confounders imperfectly shared by individuals in the pair, and in the absence of measurement error, the regression coefficient obtained from this model (the “within” estimate) will be an unbiased estimate of the causal effect of exposure on outcome [34]. In the presence of measurement error or imperfectly shared confounders the interpretation of the within estimate is more complicated, and will depend on the strength of clustering of exposure and on the reliability of the exposure measurement [24]. Using the between-within model, we fit the same two models as above: adjusted for birth year, and further adjusted for socioeconomic characteristics. Note that to achieve proper adjustment for covariates in the BW-model it is necessary to include both the individual’s and his brother’s values on the covariates [34].

All analyses were made separately for full brothers, half-brothers raised together and half-brothers raised apart. To obtain statistical tests of the difference in parameter estimates between these samples, we also analysed all samples combined, with sibling type included as an effect modifier of all other covariates.

## Results

As shown in Table 1, half-brothers were twice as likely as full brothers to have been convicted of one or more violent crimes (14.1% vs. 6.6%). Half-brothers also had lower average cognitive ability (4.6 vs. 5.1). In all three groups, cognitive ability was significantly lower among those convicted of one or more violent offences (Cohen’s d full brothers: 0.72, half-brothers T: 0.58, half-brothers A: 0.59, all p-values<0.0001). Further, we explored if general cognitive ability was associated specifically with more severe violent crimes in the combined group of full and half-brothers. The average cognitive ability score was 5.2 (95% CI 5.2–5.2) among individuals *never* convicted of a violent offence, 3.8 (3.8–3.9) among those with any violent conviction, 3.8 (3.8–3.8) among those convicted for assault, 3.4 (3.4–3.5) for those convicted for threat, and 3.6 (3.5–3.7) among individuals convicted for robbery or homicide 3.6 (3.3–3.9).

**Table 1:** Proportion of men born in Sweden 1961–1975 who were convicted of one or more violent offences 1973–2009 and mean general cognitive ability, divided by sibling type.

			Cognitive ability, mean (SD)		
	Number of pairs	Violent crime (%)	No violent conviction	Violent conviction	Cognitive ability,sibling correlation
Full brothers	119,195	6.6%	5.2 (1.9)	3.9 (1.7)	0.49
Half-brothers, reared together	8977	13.8%	4.6 (1.8)	3.6 (1.6)	0.33
Half-brothers, reared apart	12,574	14.3%	4.7 (1.8)	3.7 (1.6)	0.24

**Figure 1:**
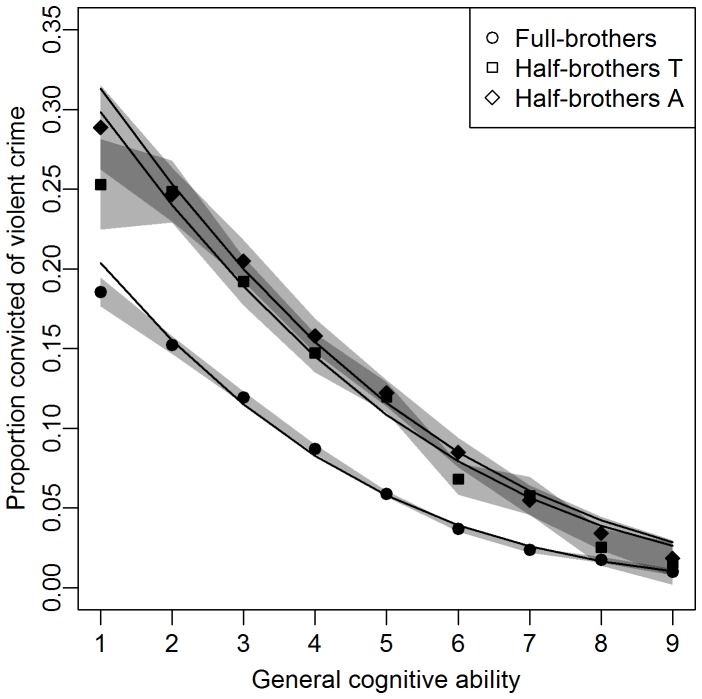
The proportion convicted of violent crime is inversely associated with intelligence. Proportion of men born in Sweden 1961–1975 convicted of one or more violent offences 1973–2009 as a function of stanine general cognitive ability (intelligence), and fit of the probit model. Observed proportions are depicted for full-brothers (filled squares), half-brothers raised together (T) and apart (A). Grey areas are based on 95% confidence intervals for these proportions, and dark grey specifically represents the overlap of confidence intervals. The lines are predicted values from linear probit regression.

Figure 1 show the proportion convicted for violent crime as a function of general cognitive ability score. Lower cognitive ability was associated with higher violent crime rates across all intelligence levels. Although not a linear relationship on the percentage scale, the linear probit model gave predictions close to the observed values, albeit slightly overestimating the proportion convicted in the tails of the intelligence distribution. Including second, third and fourth order effects of intelligence would have yielded even better predictions (including such terms were all significant at α = 0.05), but would have made sibling comparisons much more difficult to present and interpret [35]. Judging the discrepancy between expected and observed values under the linear probit model to be unlikely to have a great influence on our conclusions, we did not include any higher order effects of cognitive ability in the regression models.

The inverse association of cognitive ability and violent offending was very slightly decreased by adjustment for childhood socioeconomic variables (family income, living with single mother, urbanicity) (Table 2). Although the base rate of violent offending differed across groups, probit regression coefficients of cognitive ability on violent offending were not significantly different (full- *vs.* half-brothers T p = 0.34, full- *vs.* half-brothers A p = 0.09, half- T *vs.* half-brothers A p = 0.71). The within-pair coefficients were all lower than the unpaired regression coefficients. The coefficients among full-brothers deceased from −0.19 to −0.10, for half-brothers T from −0.18 to −0.13 and for half-brothers A from −0.18 to −0.16. The groups were significantly different in within-pair coefficients (full- *vs.* half-brothers T p = 0.01, full- *vs.* half-brothers A p<0.0001, half- T *vs.* half-brothers A p = 0.04). Part of this attenuation is likely caused by measurement error, shown to have a greater attenuating effect on within-pair effects as the pair correlation in exposure increases [24], [36]. Briefly, the within-pair association is free from variance shared by the pair, while variance that is not shared is preserved. If measurement error is random, the pair will not share it, so when removing shared sources of variance the percentage of exposure variance caused by measurement error will increase. If 20% of exposure variance were due to measurement error, the unpaired estimate would be attenuated by 20% compared to the causal effect. However, within-pair, measurement error would account for more than 20% of the variance, and the within-pair estimate thus further attenuated. In the absence of confounding, the expected attenuation may be calculated as a simple function of the reliability of the measurement and the observed sibling correlation in exposure [24]. Unfortunately, we have failed to find any publically available estimate of the reliability of the since long replaced SEB80, but as argued in the Methods section it is unlikely to be lower than 0.8, and perhaps more likely closer to 0.9, which is also similar to other validated intelligence tests; for example, the WAIS-IV reports a reliability of above 0.9 [37]. As an illustration, Table 3 shows the expected within-pair estimates calculated over a range of hypothetical SEB80 reliabilities.

**Table 2:** Probit regression of general cognitive ability on violent offending (1973–2009) in Swedish men born 1961–1975, ordinary unpaired analysis and within sibling-pair, stratified by sibling type.

	Unpaired		Within-pair	
	Model 1^A^	Model 2^B^	Model 1^A,C^	Model 2^B,C^
Full brothers	−0.19 (−0.19; −0.18)	−0.18 (−0.18; −0.17)	−0.10 (−0.11; −0.09)	−0.10 (−0.11; −0.09)
Half-brothers reared together	−0.18 (−0.19; −0.17)	−0.17 (−0.19; −0.16)	−0.13 (−0.15; −0.11)	−0.13 (−0.15; −0.11)
Half-brothers reared apart	−0.18 (−0.19; −0.17)	−0.17 (−0.19; −0.16)	−0.16 (−0.18; −0.14)	−0.16 (−0.18; −0.14)

**Notes:** Numbers are probit regression coefficients with 95% confidence interval within brackets.

A)Adjusted for birth year.

B)Adjusted for birth year and childhood socioeconomic variables: growing up with single mother, family income, and urbanicity.

C)Within-pair adjustments also included brother’s corresponding covariates.

**Table 3:** Expected within-pair regression coefficients of general cognitive ability (intelligence) on violent offending, divided by sibling type.

	Intelligence test reliability					
	1	0.9	0.8	0.7	0.6	0.5
Full brothers	−0.19	−0.17	−0.14	−0.11	−0.07	−0.01
Half-brothers,reared together	−0.18	−0.17	−0.16	−0.14	−0.12	−0.09
Half-brothers,reared apart	−0.18	−0.17	−0.16	−0.15	−0.14	−0.12

**Note:** Figures were calculated based on the assumption that the observed birth-year adjusted coefficients depicted in Table 2 are correct, and there is no confounding. Reliability is unlikely to be below 0.8.

As seen in Table 3, the reliability needs to be improbably low, between 0.7–0.6, to completely explain our observed within-pair estimates. Hence, within the range of likely reliabilities, 0.9–0.8; the observed within-pair coefficients for full brothers and half-brothers T (as seen in Table 2) are lower than the coefficients we would expect in the absence of confounding. The expected values lie outside the 95% CIs of the observed associations, so this difference is unlikely to be due to chance. For half-brothers A, the observed coefficient is not significantly different from the value expected under no confounding. Hence, the association of low cognitive ability and violent offending seems partly explained by confounding factors that are progressively shared by half-brothers A, half-brothers T and full brothers.

## Discussion

Following a large male total population sample over 35 years, we found an inverse association between cognitive ability measured in early adulthood and having been convicted of one or more violent crimes. The association decreased only marginally when adjusted for measured childhood socioeconomic variables. Further, it was attenuated but remained significant within brothers, indicating some confounding by factors shared by brothers growing up together and more so by full brothers than by half-brothers. However, this confounding could only partly explain the association, and does not disprove the widespread hypothesis that lower cognitive ability has a causal effect on criminal behaviour.

### Interpretation of Sibling Comparisons

In psychology and epidemiology, the within-pair coefficients from sibling comparisons such as the between-within model or co-twin control designs have often been described as adjusted for shared familial (genetic and environmental) confounding [38], [39], so that the within-pair estimate among full siblings could be interpreted as adjusted for all confounding by shared environment factors, and half of all confounding from genetic factors. However, recent work [24] and a rediscovery of research in econometrics [36], [40] suggest that this may not be a completely accurate interpretation. A “traditional” interpretation of the results from this study, with within-pair coefficients progressively lower among half-brothers A, half-brothers T, and full brothers, may be that we found evidence for both environmental and genetic confounding. And possibly, by extrapolating to what one might find in monozygotic twins, that the pattern suggests that the bulk of the association could be attributed to genetic confounding. Unfortunately, however, this interpretation may not be entirely correct.

The interpretation of sibling comparisons have been discussed in detail elsewhere [24], [40]. In short, the problem with the traditional interpretation of sibling comparisons is that only sibling pairs that differ in the exposure variable (cognitive ability) will influence the within-pair estimate. This leads to an implicit selection of pairs that, despite the fact that siblings tend to be similar in cognitive ability, are for some reason different on this trait. In turn, this means that these pairs, though completely similar on factors all siblings share, will tend to be more different on non-familial causes of cognitive ability than two randomly selected individuals with the same cognitive ability difference.

While this selection would indeed remove confounding by factors shared completely by the pair, other confounding may actually be increased in sibling comparisons, compared to an unpaired estimate. It turns out that the relative sibling correlation in exposure compared to sibling correlation in confounder determines whether a sibling comparison will increase or decrease confounding compared to non-sibling comparisons. Specifically, confounding by factors that are more correlated within-pair than the exposure would be reduced, confounding by factors less correlated than the exposure would be increased, and confounding by factors equally correlated as the exposure would not be influenced by the within-pair estimation [24]. The selection described in the previous section also has the consequence that effect attenuation due to measurement error will be stronger for within-pair coefficients than for unpaired estimates, meaning that we would generally expect within-pair estimates to be lower than unpaired estimates, even in the complete absence of confounding [24], [41].

In the present case, all within-pair coefficients were lower than the unpaired effects. What could explain these decreases? First, it’s possible that they simply reflect increased attenuation due to measurement error. We checked this by calculating the expected within-pair coefficients for the different brother groups, as a function of the reliability of the SEB80 and under the assumption of no confounding, and comparing this to the observed values. The reliability of the SEB80 is likely to be in the range of 0.9 to 0.8. Within this range, it is possible that measurement error would completely explain the within-coefficient among half-brother A pairs, but the coefficients among half-brother T pairs and full brothers are significantly lower than expected. Though attenuation due to measurement error is certainly present, some additional factor(s) must explain the additional decrease observed within half-brothers T and full brothers.

Second, it is theoretically possible that our sibling comparison increased confounding from factors correlated less strongly in brothers than cognitive ability is. Compared to many other traits, cognitive ability has an exceptionally high sibling correlation, so this may not be completely unexpected. But if *increased* confounding were to *decrease* the association, then the confounder would have to result in a positive association between high intelligence and high propensity to violent offending. Although some studies from clinical settings indicate that there may be such an association among individuals with psychopathic personality [42], [43], such confounding seems unlikely to have a great influence on the general association of cognitive ability and violent offending.

Third, and the motive for using sibling comparisons, we may be removing confounding from factors with higher sibling correlation than cognitive ability. Any such confounder would need to have a sibling correlation similar to cognitive ability among half-brothers A (ρ_IQ_ = 0.24, Table 1), a stronger correlation than cognitive ability among half-brothers T (ρ_IQ_ = 0.32), and an even stronger correlation than cognitive ability among full brothers (ρ_IQ_ = 0.49). It seems to us that only factors *shared extensively by brothers growing up together* would fit this description. That full brothers raised together would share some factors more strongly than half-brothers raised together does not seem unlikely. Full brothers may, for instance, share influence from fathers to a higher degree, even when half-brothers are raised in the same home. Full brothers may also be closer in age and thus share cohort or period effects more. It is also likely that half-brothers raised apart would still be correlated, though more weakly, on such factors. Although raised in different homes, they still share one parent and will probably live under similar socioeconomic circumstances.

We conclude that the results of our sibling comparisons are coherent with a combination of attenuation due to measurement error, and a reduction in confounding by familial factors shared by brothers raised together. This may initially seem to contradict our adjusted analysis in Table 2, suggesting only a very modest reduction in the association of intelligence and offending when adjusted for childhood socioeconomic variables. However, we would not expect more than a minor reduction such as this if confounding is only partly causing the observed association, and there was residual confounding due to imperfect measures of the true confounding variables.

Lacking appropriate additional measures, we are not able to specifically test any hypotheses on what these shared factors may be. From the pattern of effects, we could speculate that they are perhaps related to parenting practices, including intellectual or pedagogic stimulation, or even abuse/neglect. Both cognitive ability and violent criminality are heritable [26], [27], [44], so individuals with lower cognitive ability and convictions for violent offending are at increased risk of having parents with the same characteristics. For instance, parents with weaker cognitive resources may be less adept at providing a stable rearing environment for more aggressive children.

Half-brothers raised together are more likely to be maternal half-brothers, meaning that they not only share more family-level risk factors than half-brothers raised apart, they also share more prenatal risk factors. We attempted to address this by also analysing full-brothers raised apart, and stratifying the half-brothers T and A by sharing mother vs. father. Unfortunately, due to lack of power we were not able to draw any firm conclusions from this analysis (data not shown). Similarly, full brothers raised apart would have made an interesting comparison group, and perhaps allowed for some separation of genetic and environmental sources of confounding. However, with only 930 such pairs, the within-pair estimate was −0.14 (95% CI −0.21; −0.07). Although in the direction predicted if confounding were shared more strongly among brothers reared together, the estimate is very imprecise.

Importantly, sibling comparisons are not able to separate the effect of cognitive ability from factors with a similar correlation across brothers. Since full-brothers have a correlation of 0.49 (see Table 1) on cognitive ability, the within-full-brothers estimate will not be adjusted for confounders with correlation near 0.5 in full-brothers, such as any direct additive effects of genes. For instance, it has been argued that the association may be due to other psychological traits, such as executive functioning or impulsivity [15]. Since these factors have heritabilities similar to that of intelligence [44]–[46], they are likely to have a similar pattern of correlation over half- and full-brother relations, and may potentially explain the association of cognitive ability with criminal offending.

### Methodological Considerations

By adjusting for birth year as a categorical variable, we accounted for cohort effects on crime rates, and largely for differences in time-at-risk for being convicted of a violent crime. However, we did not account for censoring due to deaths, emigration, or incarceration. Individuals who died or migrated had higher cognitive ability scores (5.7 *vs* 5.1), but despite their reduced time-at-risk they were at an increased risk of being convicted of a crime (8.7% *vs* 6.8%). However, as shown in the Methods section, only 1.5% died and 6.1% emigrated before end of follow-up. Thus, even though we may suspect the censoring to result in slight overestimation of the association of low cognitive ability and criminality, it cannot have any large impact on our results.

Despite previous claims that registered and self-reported delinquency are very similarly associated to low cognitive ability [2], we remind the reader that our measure of violent offending is based on court convictions. Being reported, apprehended, and convicted are necessarily parts of this phenotype. We consider it likely that part of the relatively strong association we find is due to that violent offenders with lower cognitive ability are easier to identify or might be more truthful, less deceptive and even fare worse during interrogation and court proceedings.

### Conclusions

If cognitive ability influences the propensity for violent offending, this opens an opportunity for selective prevention of crime through interventions addressing children with weak cognitive resources. However, before considering such costly and potentially labelling strategies, it is prudent to ascertain that the association is truly causal and not spurious. Based on the present study, we conclude that most of the association is not due to confounding by childhood environment.

Men convicted of violent crime had more than a standard deviation lower cognitive ability than those without such convictions. Beyond the discussion of a potentially causal effect of lower cognitive ability on violent offending, this is clear evidence that violent individuals managed in courts, prison and probation, and forensic psychiatric services, on average have weaker cognitive resources. Although probably unsurprising to practitioners in these settings, it seems important that policy makers and managers in different parts of the judicial system appropriately recognise this. Further, successful participation in treatment programs aimed at reducing criminal recidivism may also be affected unless varying individual responsivity is appropriately accounted for as suggested in the Risk, Needs and Responsivity model for effective correctional treatment [47]. Indeed, while avoiding an overly deterministic position, ignorance regarding the association of low cognitive ability and criminal offending might risk leading to too cognitively demanding treatment interventions or unrealistic expectations of the potential and efforts needed to acquire higher education among former violent offenders.
